# Behavior change: from neurobiology to behavioral change theories and back – part 2

**DOI:** 10.1055/s-0046-1827212

**Published:** 2026-08-31

**Authors:** Fabiano Moulin de Moraes, Conrado Regis Borges, Marcelo Youngblood, Felipe Chaves Duarte Barros, Nancy Huang, Marcos Christiano Lange

**Affiliations:** 1 Universidade Federal de São Paulo, Faculdade de Medicina, São Paulo SP, Brazil.; 2 Faculdade Sírio-Libanês, São Paulo SP, Brazil.; 3 Universidade Estadual de Ponta Grossa, Faculdade de Medicina, Ponta Grossa PR, Brazil.; 4 Universidade de São Paulo, Faculdade de Medicina, São Paulo SP, Brazil.; 5 Universidade Federal do Paraná, Faculdade de Medicina, Curitiba PR, Brazil.

**Keywords:** Health Behavior, Behavior Therapy, Motivation, Preventive Medicine, Lifestyle Medicine, Health Education, Neurobiology

## Abstract

**Background:**

Behavior change remains challenging despite a vast body of evidence linking health behaviors with several health outcomes. Over the past few decades, various health behavioral change theories and techniques have emerged, leading to significant advances in the field. However, although some existing theories provide competent models for structuring approaches, they do not provide a unified neurobiological foundation.

**Objective:**

To outline the connections between neuroscientific foundations in Part 1 and behavioral change theories in Part 2 and propose an overarching neuroscientific perspective.

**Methods:**

A narrative review of the relevant history and background of the most widely adopted behavior change theories and integrates them into the proposed framework of the Behavior Change Resource Model described in Part 1.

**Results:**

A structured overview of selected theories was performed. The main theories and techniques described were Social Cognitive Theory, Self-Determination Theory, Motivational Interviewing, Behavioral Activation, Transtheoretical Model, Cognitive Behavioral Therapy, Mindfulness Meditation, Positive Psychology, and Health and Lifestyle Coaching. Finally, their relation to motivation science and the Behavior Change Resource Model was detailed.

**Conclusion:**

By bridging the Behavior Change Resource Model (Part 1), and an array of related theories and techniques (Part 2) it was possible to provide a conceptual perspective to further support and propel the scientific understanding of human behavior, especially towards keeping and expanding health through lifespan and across society.

## INTRODUCTION

Part 1 of this review described the neuroscience of behavior change and maintenance from neuroanatomy to neurophysiology and, finally, to a neuroscientific-based motivational theory called the Behavior Change Resource Model.

The main goal of part 2 is to detail the history and background of various theoretical perspectives that study behavior-changing processes and are the foundations of the proposed model, such as: Social Cognitive Theory (SCT), Self-Determination Theory (SDT), Motivational Interviewing (MI), Behavioral Activation (BA), Transtheoretical Model (TTM), Cognitive Behavioral Therapy (CBT), Mindfulness Meditation (MM), Positive Psychology (PP), and Health and Lifestyle Coaching (HLC). Then, the purpose is to connect them to the specific stages of motivational engagement they emphasize, and the strategies they employ, to find a common language among them.

## METHODS

The present narrative review aims to summarize existing literature on the research question in a comprehensive manner. This methodology was chosen to explore this broad and emerging topic that requires interpretive synthesis.

To identify relevant articles, the following online databases were used: Scopus, PubMed, and Web of Science. Additionally, the citations and reference lists of the retrieved relevant reviews were screened for any additional relevant publications. The search was conducted from March 2025 to October 2025. The following inclusion criteria were used for each study:

- Published in English as peer-reviewed empirical research;
- Research methods used conducted qualitative research, quantitative research, meta-analysis, and reviews;
- The use and the features of any behavior change technique or techniques in humans, healthy controls or those with any medical conditions.

The database was queried to search for the following terms in the articles' titles, abstracts, and keywords: *behavior change* OR *behavior change technique* OR *health behavior* OR *behavior change theory* OR *behavior change theory interventions* OR *The Behavior Change Resource Model* OR *Motivation* AND *Social Cognitive Theory* , OR *Self-Determination Theory* , OR *Motivational Interviewing* , OR *Behavioral Activation* , OR *Transtheoretical Model* , OR *Cognitive Behavioral Therapy* , OR *Mindfulness Meditation* , OR *Positive Psychology* , OR *Health and Lifestyle Coaching* .

## VIEWS OF HUMAN NATURE

There is no universal taxonomy of motivation and behavior change, 1 2 but it is possible to didactically describe the schools of thought as humanist (mainly concerned with intrinsic motivation), behaviorist (mainly concerned with extrinsic motivation), and mixed (related to both). 3 Motivation represents the desire to accomplish a task, coupled with the eagerness and resolution to persist towards that goal. Intrinsic motivation refers to what emerges internally as interests and desires, while extrinsic motivation refers to what is elicited by external factors like penalties or incentives. 4 It is important to emphasize that this taxonomy is intended solely to provide conceptual background and is not exhaustive.

In the subsequent sections, a roadmap of these traditions of thought (Humanist, Behaviorist, and Mixed) alongside their respective models of behavior change is detailed.

## HUMANIST APPROACHES

The Humanist school posits that humans are naturally oriented toward growth and well-being, and they actively engage with their environment to achieve these ends. 5 6 7 8 9 10 Thus, motivation arises from within the individual and can be either fostered or thwarted by the environment. From this perspective, the key to intervention is to assist clients in drawing on their own motivation and fostering the types more likely to sustain behavior in the long term. While different theoretical approaches under this broad umbrella indeed emphasize different ways to achieve this, they all broadly view the client or patient as a partner in the behavior change process.

Early and fundamental theories developed through the 1970s and 80s included SCT and SDT 11 12 13 14 followed by MI. 6 15

### Social cognitive theory (SCT) and self-determination theory (SDT)

Proposed by Albert Bandura, the SCT asserts that individuals acquire new behaviors through both trial and error and by copying the behavior of others (role models) and the environment. Thus, the performance of new behaviors is enhanced by outcome anticipations, self-efficacy, and identification. 13 14

**Figure 1:**
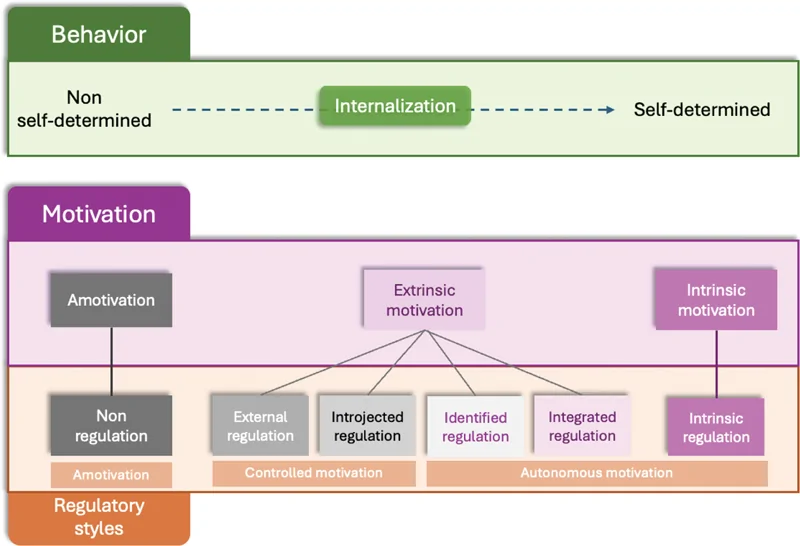
Source: Adapted from Ryan and Deci. 13 Self-determination theory (SDT) framework of motivations for behavior.

The SDT was described by Deci & Ryan in the early 1980's as a humanistic, person-centered approach to motivation. 16 The premise of SDT is that motivation arises from within a person, rather than being given to them or imposed on, and that supporting the persons' basic necessity for autonomy (being the causal agent of one's life), competence (the ability to master skills that matters to oneself), and relatedness (feeling connected to others) best enhances the initiation and maintenance of behavior change. 17 This approach focuses on helping clients develop their own value for and interest in health behaviors while making plans that support changing goals from initiation through maintenance, guiding the client from amotivation or extrinsic motivation to intrinsic ( Figure 1 and Table 1 ). 9 18

**Table 1:** The three basic psychological needs in SDT: Relatedness, Autonomy, and Competence. Adapted from 13

Basic psychological need	Definition	How needed is supported
Relatedness	The need to feel connected to and valued by important others. The sense that one is significant and worthy.	Unconditional positive regard. Support from the practitioner and from others outside of treatment.
Autonomy	The need to feel willingly engaged in their behaviors and a sense of ownership of their actions.	Understanding client's perspective. Active exploration of clients' thoughts and emotions. Develop personally relevant health behaviors.
Competence	The need to feel effective and capable and develop a sense of mastery over one's behavior.	Meeting clients at their skills and abilities. Translating planning into practice. Develop realistic expectations and coping with inevitable disappointments.

Then, SDT has been applied in a wide range of conditions with solid evidence, serving as a foundation for other behavior change theories, such as MI and HLC. 19 20 21

### Motivational interviewing (MI)

The MI method, 6 15 which originated in addiction treatment, is a solid concept for encouraging motivation to change in patients who are unwilling or ambivalent about making changes. This method combines various evidence-based approaches, including cognitive psychology, social psychology, and SDT.

It has a few principles. The first is that the benefits of healthy behaviors (such as a better diet or improved fitness) are countered by the downsides of behavior change (for example, loss of social ties, significant pain, or effort). 22 The second is that people with unwanted behaviors (for example, unhealthy diet, smoking) have different levels of readiness for behavior change. In this context, individuals with problematic behaviors are not unmotivated to change, but rather ambivalent about it. These behaviors represent, at least to some extent, their self-concepts or values, creating reasons against change. If this ambivalence is not recognized, well-intentioned advice is perceived as an attack on their freedom of choice (autonomy), often leading to noncompliance. 23

Thus, MI is defined as “a person-centered, goal-oriented style of communication with a particular focus on expressions of change”. 22 The goal is to increase personal motivation for and commitment to behavior change by eliciting and intensifying a person's reasons for change in an atmosphere of acceptance and empathy”. 6 This model works with four major processes, intertwined with four core skills ( Table 2 ). 24

**Table 2:** Processes and skills of MI 19

Processes	
Engaging	Building a foundational relationship, based on empathic, nonjudgmental and interested listening.
Focusing	Developing a clear direction and goal. Finding a priority.
Evoking	The interview becomes goal oriented. Motivation to change is incited and “change talk” is reinforced.
Planning	Formulate a concrete plan and “engagement talk”.
Skills (OARS)	
Open questions	Questions that cannot be answered with “yes” or “no” answers. For example: “What concerns do you have?”
Affirmations	Validate and acknowledge positive attributes, efforts, strengths, etc.
Reflections	Demonstrate active listening and invite exploration. For example: “I see that this upsets you…”
Summaries	Bundle concluding reflections, invite elaboration, conclude the session or check clarity For example: Let me summarize what I heard you say, please let me know of it correct…”

Abbreviations: MI, motivational interviewing; OARS, open questions, affirmations, reflections, summaries.

Since the approach was first developed, the number of MI-specific publications has increased exponentially, to the point that there are now more than 1,300 randomized trials and around 150 reviews on its effectiveness in various behaviors and target populations. 15 24 25

## BEHAVIORIST APPROACH

The behaviorist school, rooted in behaviorist tradition, proposes that individuals behave in response to environmental contingencies. 26 27 According to this perspective, the key to intervention lies in identifying the “right” reinforcements (rewards) that will increase desired behaviors and the “right” punishments that will extinguish undesirable behaviors. 26 28 Therefore, behavior change is best understood as a function of learning these contingencies and consistently applying them to sustain the behavior. 1 2

From this perspective, BA, since its formulation, has become the bedrock of the behavior change model. 27

### Behavioral activation (BA)

Initially created for depression treatment in the 1970s, 26 BA is now widely used for behavior change in other settings, 27 including with individuals with high risk for diseases such as dementia, stroke, and cardiovascular diseases. 29 30 Although there are differences between studies and protocols, the following components of care are typically included: activity monitoring, assessment of life goals and values, activity scheduling, skills training, contingency management, and procedures targeting avoidance. 27

Activity monitoring is a first step that describes the behavior's details (frequency, intensity, or other relevant features) to be changed. Whether written in charts or other media, it serves as a necessary base for change, and sometimes it is enough to trigger it.

Assessment of life goals and values evaluates the person's goals and values to direct activation assignments. 31 Usually, the client receives a list of domains (e.g., relationships, family, physical/health issues, education/training, and spirituality) and is guided by the professional to identify their most relevant values, culminating in detailed statements. In this perspective, values act as reinforcers, guiding the client toward a predefined positive reinforcer. 31

Activity scheduling (AS) is a hallmark of treatments for behavior change and depression. It increases contact with positive reinforcers in the environment, considering the person's possibilities and values. It adds cues or prompts (such as homework assignments) into the client's environment to elicit the targeted behavior at a higher rate, including what, when, where, and how.

Skills training (ST) interventions are used when a person is unable or lacks the knowledge to engage in effective behavior. These include both social and nonsocial skills, such as interpersonal skills, assertiveness, and problem-solving.

Contingency management (CM) addresses contexts in which approximations towards the desired behavior are ignored, punished, or not reinforced by the environment, or when undesired behavior is sustained by positive or negative reinforcement. Essentially, the environmental consequences are problematic and need to be adjusted to support proper behavior.

Finally, methods targeting avoidance are crucial for behavior change, as most changes create discomfort, whether it involves increasing or reducing a habit. The idea is to help clients “act according to a goal rather than according to a feeling” or work from the “outside in”. A summary of this technique is described through the acronym TRAP, where T stands for trigger, R stands for response, and AP stands for avoidance pattern. 32 By increasing alternative coping (AC) behaviors, the goal is to replace the AP of TRAP with an AC, creating a well-timed phrase “Get out of the TRAP and get back on TRAC(K)”. 32 The BA model remains relevant and serves both as a foundation for the CBT and PP movement and as a counterpart. 33

### Mixed approaches

The mixed school was developed drawing from both lines of thought (humanist and behaviorist), either in response to certain criticisms or from ramifications within each school, widening our perspective on behavior, chance, and human motivation. 34 35 Several approaches to behavioral change may be classified as mixed because they align with both of the previously described schools of thought.

### Transtheoretical model (TTM) of behavioral change

The TTM of behavioral change is one of the most commonly used frameworks for behavioral change in medical and psychological literature. 10 This model states that changing behavior is a process rather than a single event, characterized by a sequence of stages and processes through which individuals progress toward adopting adaptive behaviors. It implies that different people are in varying stages of change (SC) and readiness, and that lapses and relapses can occur.

**Figure 2:**
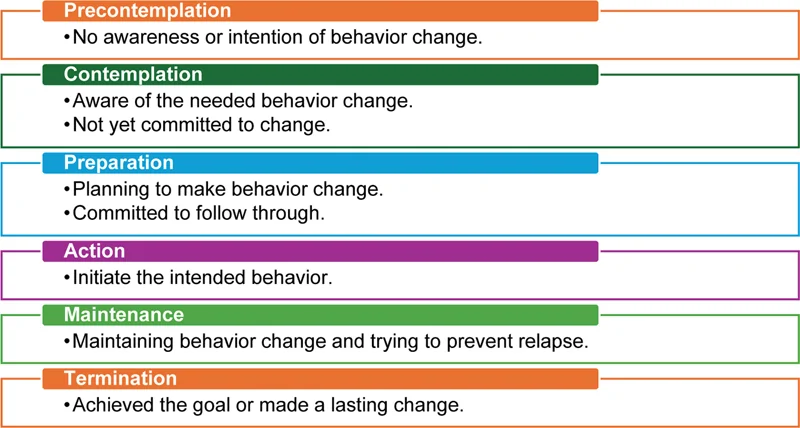
The six stages of behavior change model. **Source** : Adapted from Austin. 33

**Figure 3:**
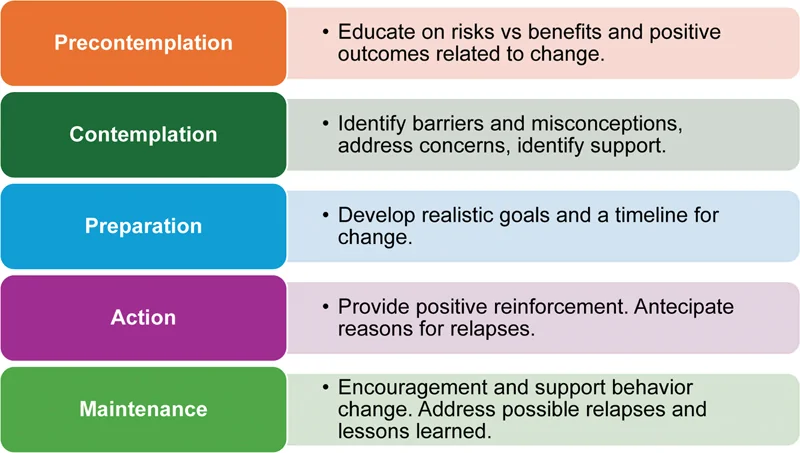
Strategies used in each stage to progress behavior change. **Source** : Adapted from Austin. 33

In this process, people typically progress through five stages: precontemplation, contemplation, preparation, action, and maintenance ( Figure 2 ). Each stage is clearly defined and offers opportunities to use specific techniques that help individuals begin, sustain, and advance through it ( Figure 3 ). 36

The processes of change (PC) describe how people change as they progress through the stages ( Table 3 ). These are strategies and techniques that people use to change or modify their behavior and can be divided into two categories: cognitive (thinking, attitudes, awareness) and behavioral (actions) processes. 37

**Table 3:** Process of change in TTM focused on PA, as an example 34

Cognitive processes of change	
Consciousness raising	Increasing knowledge about the benefits of PA.
Dramatic relief	Awareness of health risk of lack of PA.
Environmental reevaluation	Analyze how physical inactivity affects your relations and environment.
Social and self-liberation	Show different ways to be active. Personalize.
Decisional Balance	Perceived benefits and barriers of engaging in regular PA.
**Behavioral processes of change**	
Counter conditioning	Change inactive habits for healthy ones.
Helping relationship	Social support to facilitate change.
Reinforcement management	Reward yourself for being active.
Self-liberation	Commitments and planning.
Stimulus-control	Use of the environment as a stimulus to maintain PA levels.

Abbreviations: PA, physical activity; TTM; transtheoretical model.

It is important, however, to note that the core assumption of sequential stage progression in TTM has been questioned in the literature because of many concerns about the validity of stage assessments. 38

### Cognitive behavior therapy (CBT)

The CBT is a psychosocial intervention originally designed to treat mental disorders, such as depression, and has been recently adapted to improve health behaviors. 39 It combines the core principles of behavioral and cognitive psychology, challenging unhelpful cognitive distortions (e.g., thoughts, beliefs, and attitudes) and behaviors, while improving emotional regulation and developing personal coping strategies to address current problems. 12 Numerous studies have shown that CBT and its various subtypes, such as Acceptance and Commitment Therapy, are well-suited to support behavior change across diverse scenarios. 40

### Mindfulness meditation (MM)

Meditation is a form of mental training that aims to improve an individual's core psychological abilities, including attentional and emotional self-regulation. 41 It encompasses a family of complex practices, such as MM, mantra meditation, transcendental meditation, and others.

Among these practices, MM—often described as nonjudgmental attention to present-moment experiences—has received the most attention in neuroscience research over the past two decades. 42 It includes at least three closely interacting components that form a process of enhanced self-regulation: enhanced attention control, improved emotion regulation, and altered self-awareness (including diminished self-referential processing and increased body awareness). 43 44

A growing body of evidence indicates that mindfulness-based interventions (MBIs) effectively reduce harmful health behaviors by catalyzing chronic disease self-management and health behavior change, and improving physical and mental health outcomes, whether alone or combined with other techniques such as CBT. 8 43 44

### Positive psychology (PP)

The positive psychology movement emerged in the wake of psychology's emphasis on mental disorders while ignoring mental health and well-being. 45 It quickly gave rise to the development of PP interventions (PPI)—an umbrella term for activities that promote positive thoughts, emotions, and behaviors, with the long-term goal of contributing to psychological growth and well-being. The PPI ranges from a single activity, such as gratitude journaling or performing an act of kindness, to a multi-component intervention, such as the positive psychotherapy program 46 47 48 and the positive emotion, engagement, relationships, meaning, and achievement (PERMA) model.

This model describes a range of PPIs that foster positive emotions, including not only happiness but also hope, love, and gratitude. Engagement, self-consciousness and complete absorption in an activity. Relationships, including all the various interactions of individuals with family members, friends and their community at large. Meaning, a sense of belonging and serving something greater than ourselves. Lastly, accomplishments, a sense of achievement, mastery, and competence. Recently, the PERMA model added another letter, V, for vitality, emphasizing the importance of caring for one's physical health. 46 47 48

Over the last four decades, evidence supporting PPI's effectiveness has grown exponentially, ranging from mental health treatments to strategies to enhance the efficacy of behavior change techniques. 5 46 47 49 50

### Health and lifestyle coaching (HLC)

The HLC model is a “patient-centered approach in which patients identify their goals, work towards achieving them, and self-monitor their behaviors to enhance accountability with the support of a coach”. 7

The coach is a healthcare professional trained in behavior change theory, motivational strategies, and communication techniques. These tools help patients develop intrinsic motivation and acquire skills to create sustainable change, leading to improved health and well-being. 51 52 The main theoretical underpinnings of health coaching include CBT, TTM, SDT, MI, and PP. 28 51 53

Various systematic and literature reviews, ranging in size from 13 to 219 studies, including either randomized controlled trials (RCTs) or mixed quantitative and qualitative methodologies, have evaluated HLC outcomes across a diversity of conditions and diagnoses, such as stroke, dementia, cancer, heart disease, diabetes, obesity, hypertension, and wellness, among others. These reviews indicate that HLC is beneficial for physiological, behavioral, and psychological conditions, as well as social life outcomes. 7 53 54 55 56 57 58

## A BROAD VIEW OF NEUROBIOLOGY AND ITS RELATIONSHIP WITH THE BEHAVIOR CHANGE RESOURCE MODEL

As shown previously, the neurobiology of behavior and behavior change sciences are vast research fields with several overlapping or even contrasting themes. To provide a framework for organizing these theories, we present Table 4 , which links the aforementioned theories with the engagement stages for which they are best suited, as well as to the types of resources they offer within the Behavior Change Resource Model described in Part 1. The underlying premise of this table is that, once the relevant engagement stage has been identified—whether at the individual, group, or community level—it becomes possible to select and apply a range of techniques aimed at enhancing ability, motivation, and competence, or at implementing the environmental modifications necessary to support progress toward the desired goal. This table presents a conceptual heuristic mapping by the authors, based on theoretical plausibility, and requires further validation through future research.

**Table 4:** Behavioral change theories and their potential uses in distinct engagement stages

Theories classification	Behavioral change theories	Engagement stages							Resources		
		Non engagement		Motivational engagement		Executive engagement			Ex	Af	Re
		U	A	C	P	I	CA	M			
Humanist	SDT	X	X	X	X					X	
	SCT	X	X	X	X					X	
	MI	X	X	X	X	X				X	X
Behavioral	BA				X	X	X	X	X	X	X
Mixed	TTM	X	X	X	X	X	X	X	X	X	X
	CBT	X	X	X	X	X	X	X	X	X	X
	MM		X	X			X	X		X	X
	PP	X	X	X	X	X				X	
	HWC	X	X	X	X	X	X	X	X	X	X

Abbreviations: SDT, self-determination theory; SCT, social-cognitive theory; MI, motivational interview; BA, behavioral activation; TTM, transtheoretical model of change; CBT, cognitive-behavioral therapy; MM, mindfulness meditation; PP, positive psychology; HWC, health and wellness coaching; U, unawareness; A, awareness; C, commitment; P, planning; I, initiation; CA, continued action; M, maintenance; Ex, external resource; Af, internal-affective resource; Re, internal-reflective resource.

Notes: Each “X” indicates a plausible conceptual link between the detailed theories, engagement stages, and resources from the Behavior Change Resource Model, as assessed by the authors.

This review, including Parts 1 and 2, describes the neuroscience and psychology of health behavior change and maintenance, ranging from neurophysiology to some of the most widely adopted behavior change techniques, and concludes with a perspective that incentivizes iterations between theories and practices from a neuroscientific framework, the Behavior Change Resource Model ( Table 4 ).

It should be clear that this integration, although intended to create a foundation, is conceptual and theory-driven, rather than empirically validated. The bridging, thus, of high-level psychological theories with low-level neurobiological systems remains a worthy scientific pursuit. Further investigation is needed to assess this model's validity and applicability across different settings, and to minimize arbitrariness related to its heuristic and theoretical nature.
